# Surgery

**Published:** 1908-12

**Authors:** James Swain


### SURGERY.

**The radical treatment of carcinoma of the bladder** is of recent origin, and the results so far obtained are not so good as those effected by operation for cancer in other parts of the body. Berg,[2] in an excellent paper on the subject, explains this partly by the difficulties which exist in dealing with this organ, and partly by the want of appreciation of certain pathological facts.

The modern ideas of a successful operation for carcinoma demand that the affected lymphatics and glands, as well as the primary mischief, should be removed; but this point has been largely overlooked in dealing with the bladder.

Klebs[3] stated in 1870 that carcinoma of the bladder is always secondary to disease of the prostate, rectum, or uterus, and that in women, at least, a primary carcinoma of the bladder cannot

[2] *Ann. Surg.*, 1908, xlviii. 355. [3] *Handb. d. path. Anat.*

occur. This question has been carefully considered by Mandlebaum,[1] who found "that the papillary and the flat or squamous-celled carcinomata do occur as primary tumours in the bladder, but that the fibro- or scirrhous-carcinomata, and the adeno-carcinomata are very often, if not always, secondary to primary tumours of the prostate, uterus, or rectum." These investigations are of the greatest importance, for they show the necessity of a correct histological knowledge of the nature of the neoplasm, and suggest that many recurrences after radical operations are due to the leaving behind of an unrecognised primary focus in some adjacent organ.

These remarks have an important bearing on the extent of the operation of radical cure, and Berg[2] says the operator must not only consider how to remove the disease widely, but also how to restore or substitute the function which belongs to the urinary bladder. The latter question has not yet been finally settled, but the use of the vagina or intestine as a substitute for the bladder by implanting the ureters into them has been largely abandoned because of the danger of an ascending pyelonephritis resulting from an infection of the ureters from these structures. The attachment of the ureters to the skin, or the direct drainage of the pelvis of the kidneys in the loin (nephrostomy) are attended by similar risks of infection, and are productive of much discomfort, though they are unavoidable in some cases.

The question of the desirability of partial or complete resection of the bladder has given rise to much discussion. Raffin's[3] statistics are in favour of partial resection, for of 30 cases of total excision of the bladder for carcinoma there was a primary mortality of 56.5 per cent., whereas in 96 cases of partial cystectomy the operative mortality was only a little more than 21 per cent. Moreover, the ultimate results were better in partial excision, for 21 out of 50 cases were known to be free from recurrence from six months to six years after operation ; but of the cases that could be traced after complete excision, only 5 survived a period of six months. On the other hand, Watson considers that the operation of choice is a preliminary bilateral lumbar nephrostomy, with ligation of the ureters, followed by total cystectomy. Berg himself considers that where the disease is primary in the bladder, and limited to one part of it, a partial cystectomy, together with the removal of all lymphatics and glands, is as good a protection against recurrence as a complete extirpation of the viscus. He would, however, limit the operation to cases in which not more than a third of the bladder is involved, and preferably when the situation of the growth is on the fundus and lateral walls. When the ureteral orifice is involved in the disease, the partial cystectomy should be accompanied by reimplantation of the ureters

1 *Loc. cit.* 2 *Ass. franç. d'Urol. Proc.-verb.*, 1905, Par., 1906, I.
3 *Surg., Gynec. and Obst.*, 1907, v. 315.

into the remaining portion of the bladder. It appears possible to remove one half of the bladder without materially interfering with its function.

When, however, the cancer is diffused over the greater part of the bladder, or when there is a severe cystitis such as would increase the dangers of ascending infection if the ureters were reimplanted in the bladder, the complete extirpation should be performed, and in such cases a preliminary nephrostomy is indicated.

**Volkmann's ischæmic paralysis** is a comparatively rare affection with which every practitioner should be acquainted. As the disease is not so well known as it should be, reference may here be made to a paper by Dr. Taylor,[1] of New York, who has collected 59 cases.

In all but two of these the forearm (flexor muscles) was involved. The other two cases occurred in the flexors of the leg and foot. The large majority of cases occur in children from three to twelve years of age, probably because the circulation of their muscles is more easily disturbed. The underlying cause in all these cases is ischæmia (or, better, oxygen deprivation), induced by direct compression of the vessels and muscles, or by contusion, laceration, thrombosis, or embolism of the vessels—alone or in combination. At least 80 per cent. of the cases have followed fractures of the upper limb where splints or plaster bandages have been too firmly applied. Complete ischæmia for more than six hours continuously is almost sure to be followed by serious contracture.

At the time of injury the circulation distal to the fracture is interfered with by the displacement of the fragments and the effusion of blood. Too tightly applied dressings enhance the obstruction to the arterial supply, and also add direct pressure upon the muscle substance itself. Riedinger believes the direct mechanical pressure upon the muscles is the more important of these factors; but in either case if the pressure continues for more than six hours the muscle substance rapidly degenerates and shortens, causing the typical deformity. The muscle is replaced by connective tissue, varying in amount with the severity of the case, and with the lapse of time the cicatrix becomes firmer and shorter, and the deformity more fixed.

The nerves, at first, may or may not be affected; but later, whether primarily involved or not, they may suffer degeneration from the pressure of the contracting cicatrised muscles, and this in turn results in atrophic changes, by which they become converted into fibrous bands, which are sometimes nodular from irregular compression.

The symptoms depend upon the degree of ischæmia present,

[1] *Ann. Surg.*, 1908, xlviii. 394.

and its duration. In severe cases the patient complains of pain almost immediately after the application of the tight dressings; and if the splint is not removed the pain increases, and a marked swelling occurs in the hand and fingers, with purple discoloration and the formation of blebs. Within twenty-four hours the hand assumes the claw shape resulting from contracture of the flexor muscles, and if the dressing is not then removed gangrene is very apt to occur a short distance below the elbow.

When the mischief is fully developed the elbow is slightly flexed, the forearm pronated, the wrist slightly flexed, and the fingers strongly flexed in the claw-hand position. In the severest cases the finger-nails are driven into the palm of the hand.

When nerve damage (usually median and ulnar) is present trophic changes (blue, cold, glossy, thin skin) develop with muscular atrophy in the nerve field, in addition to loss of sensation and paralysis.

In less severe cases the symptoms develop more slowly.

The condition should not be confused with paralysis due to nerve-injury where the muscles are flacid, and contracture, when it does occur, is late in appearing and slow in development.

It is important, both for prognosis and treatment, to determine whether the nerves have been involved. If the muscle responds to both faradic and galvanic currents there is no nerve injury; if the muscle responds to galvanic, but not to faradic current, there is nerve injury; if the muscle does not respond both to galvanic and faradic currents, there is complete muscle injury, but nerve injury is not determined.

The prognosis varies with the degree of muscle and nerve damage, and also depends upon the promptness and energy of treatment.

The longer ischæmic paralysis has existed, the more difficult it is to cure. Dressings should be at once removed if the patient complains of increasing pain, and in all cases in which the primary dressing has been removed no further attempt should be made to immobilise the muscles. Massage, electricity and active and passive motion should at once be used to restore the circulation in the damaged muscles. These measures should be repeated every few hours, until the muscles are in good condition again, when attention may again be given to the fracture itself.

After the condition has manifested itself, there is a choice between non-operative and operative treatment. Non-operative treatment consists in baths, massage, electricity and passive motion. Some authors advise repeated extension of the wrist and fingers, under an anæsthetic if necessary. Non-operative treatment is tedious, and on the whole unsatisfactory. It gives no relief to compressed nerves.

Operative treatment gives quicker and more complete results. This consists in (1) tendon lengthening, or (2) resection of both bones of the forearm. Elongation of the flexor tendons is tedious, but has the advantage of not shortening the forearm ; resection of the radius and ulna is quicker, and the slight shortening causes no functional disturbance. Both operations have, however, given good results by relieving the tension, favouring voluntary contraction, and increasing the circulation and nutrition of the muscles.

In every case of nerve compression the nerves should be released, and trophic and sensory disturbances can be relieved by neurolysis, even in cases which give no hope of the return of motor power.

Regeneration cannot occur if the muscles have been completely changed into fibrous tissue, but since this condition of the muscles cannot be determined clinically, no case should be denied the benefit of the doubt and refused the operation.

The post-operative treatment consists in baths, massage, electricity and movement until function has been restored to the muscle. The object of after-treatment is to cause absorption of cicatricial tissue and regeneration of muscle tissue. Hope must not be given up for months, as these cases are invariably tedious, especially when the nerves have been involved.

James Swain.

---

